# Epigenetics of prenatal stress in humans: the current research landscape

**DOI:** 10.1186/s13148-024-01635-9

**Published:** 2024-02-02

**Authors:** Linda Dieckmann, Darina Czamara

**Affiliations:** 1 Department Genes and Environment, Max Planck Institute of Psychiatry, Munich, Germany; 2 International Max Planck Research School for Translational Psychiatry, Munich, Germany

**Keywords:** DOHaD, Fetal programming, Prenatal stress, DNA methylation, Epigenetics

## Abstract

Fetal exposure to prenatal stress can have significant consequences on short- and long-term health. Epigenetic mechanisms, especially DNA methylation (DNAm), are a possible process how these adverse environmental events could be biologically embedded. We evaluated candidate gene as well as epigenome-wide association studies associating prenatal stress and DNAm changes in peripheral tissues; however, most of these findings lack robust replication. Prenatal stress-associated epigenetic changes have also been linked to child health including internalizing problems, neurobehavioral outcomes and stress reactivity. Future studies should focus on refined measurement and definition of prenatal stress and its timing, ideally also incorporating genomic as well as longitudinal information. This will provide further opportunities to enhance our understanding of the biological embedding of prenatal stress exposure.

## Introduction to fetal programming and prenatal stress

### The concept of fetal programming

The fetal programming hypothesis, brought forward by Barker [3], originally postulated that undernutrition in the womb during pregnancy leads to reduced growth which causes a predisposition for cardiac and metabolic disorders in later adult life. This has been expanded into the more general *Developmental Origins of Health and Disease* (DOHaD) hypothesis, stating that exposure to adverse environmental events during sensitive periods of development and growth can have significant consequences on short- and long-term health [4, 14]. Short-term adaptations of the fetus to these adverse exposures include down-regulation of endocrine, metabolic or organ function to slow down growth rate and nutrient consumption [70]. This can have influences on gene expression, cell differentiation and proliferation leading to long-term, and often irreversible, changes in the structure of function of specific tissues and vital organs [5].

In fact, fetal programming has been described as *predictive adaptive response* (PAR) [30]: if the predicted environment is similar to the recent environment, the adaptation leads to an advantage. Hence, prenatal cues and developmental plasticity could influence the development of a phenotype that is adapted to the environmental conditions in later life, if this matches the early environment conditions [7]. The *match/mismatch* and *PAR hypotheses* both propose that moderate levels of early life stress can acquire resilience to renewed stress exposure later in life by preparing the offspring to better cope with a challenging adult environment [7, 83]. However, if there is a mismatch between predicted and recent environment, this may also increase risk for adverse outcomes in later life [36]. This can be illustrated with the following example: If a fetus develops in a nutrition-low environment, its metabolism adapts to this lack of nutrition. If, however, after birth, a lot of food is suddenly available, this can predispose the baby for overweight as its metabolism has not been trained for this mismatch. And in fact, children born with a low birth weight are at greater risk for developing metabolic syndrome later in life [26].

### Definition of prenatal stress

Adverse environmental exposures involved in fetal programming are often described as *prenatal stress*, but how is prenatal stress actually defined? Prenatal stress, and stress itself, are very broad terms and different studies use different definitions. Generally speaking, stress presents if we are overwhelmed with the current situation and cannot adapt to it accordingly [16]. Several concepts of prenatal stress have been brought forward, reflecting the diversity of stressors which are present during pregnancy [18]. Psychosocial stressors, such as domestic violence and changes in personal life, may require adaptive coping by the affected individual [69]. These stressors can also affect pregnant women, and their offspring, but are not specific to pregnancy. On the other hand, pregnancy-specific stress refers to worries directly related to the pregnancy itself, i.e., concerns about outcome of prenatal screenings, or infant health and development [49]. Both, psychosocial and pregnancy-specific stress, can have strong effects on pregnancy and fetal development [18].

Following these definitions, a variety of different experiences have been explored in the prenatal context including adverse life events (e.g., trauma), depression, anxiety and contextual stress (e.g., financial difficulties), interpersonal risks (e.g., violence in a relationship) and risks due to parental characteristics (e.g., substance use) [15, 67]. Furthermore, also exposures to natural disasters during pregnancy have been studied as prenatal stressors [34, 45] and, very recently, the Covid-19 pandemic has added a whole new layer of complexity onto prenatal stress experiences [84].

Another very important point is that both the presence of a stressful event per se, i.e., the objective stress, and subjective stress, i.e., how stressful this event is perceived, need to be considered. In stress theory, three different types of stress have been described: response-based, stimulus-based and transactional-based stress [50]. In the response model, Selye [86] describes stress as physiological response pattern. In the stimulus-based definition of stress, stress is characterized as a stimulus such as traumatic events that cause certain reactions [38, 53]. This can also be defined as “objective” stress as it is based on the presence of a stressful event itself but does not take into account the intra-individual variability of how people react or are affected by the stressor. In the transactional-based definition of stress [48] on the other hand, the focus is not on the presence of the stressor itself but on the individual’s perception of this stressor. This could also be termed “subjective” stress. Any of the three types can also be found with regards to prenatal stress.

Given that fetal exposure to prenatal stress accounts for around 15% of the attributable risk for adverse mental health outcomes [29], it is important to study prenatal stress and how it affects the fetus. However, apart from the complexity of how prenatal stress is actually defined, also the exact processes which mediate fetal programming are not yet fully clear. Several possibilities have been suggested: (1) excessive exposure to glucocorticoids (GCs), (2) dysregulation of the hypothalamic–pituitary–adrenal (HPA)-axis, (3) irreversible changes in organ structure, (4) genetics, (5) epigenetic changes leading to altered gene expression and (6) cellular aging and intergenerational effects (see [51] for a review).

## Scope of this review

To conclude, prenatal stress is a very important, but also very complex trait. Hence, we focus here on one specific layer of prenatal stress, namely how prenatal stress influences DNA methylation (DNAm), as one of the best understood epigenetic signatures in humans, and which consequences this might have on child health outcomes. We discuss mainly evidence from human studies but would like to point out that also animal studies support the hypothesis that prenatal stress could leave lasting signatures in DNAm (see [14] for a review). We conclude on what is important for future studies to further enlighten our understanding of prenatal stress, epigenetics and child outcome.

## How does prenatal stress influence DNA methylation signatures in the offspring?

**Fig. 1:**
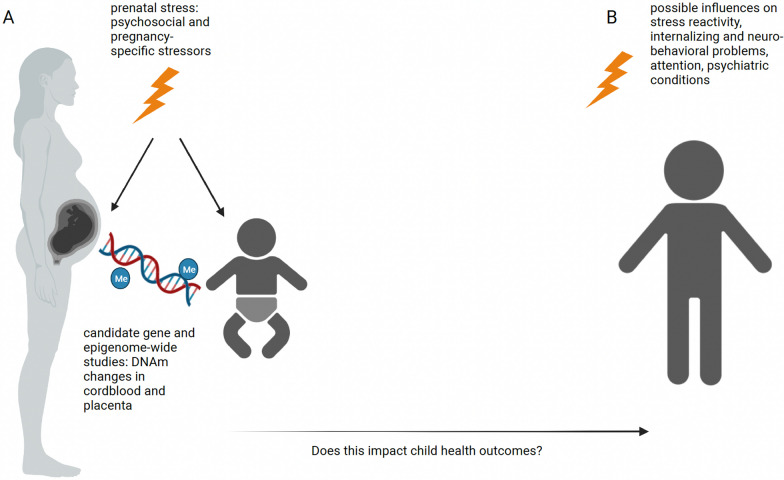
DNAm and prenatal stress. Prenatal stress can impact DNAm in perinatal tissues such as cordblood and placenta (**A**). This might have consequences on the child’s health including stress reactivity, neurobehavioral problems and psychiatric conditions (**B**). Created with BioRender.com

Epigenetic mechanisms, including DNAm, histone modifications and microRNAs, are one possible process of how prenatal stress could prime the offspring’s development (see Fig. 1A).

All of these epigenetic signatures could be influenced by prenatal stress and in turn regulate gene expression. Given that DNAm can be measured relatively straight-forward using large-scale methylation array techniques, it is the most widely studied and, so far, best understood, epigenetic mark in humans. As DNAm is tissue-specific [103], it should be mentioned that most studies assessed DNAm in buccal cells, saliva and blood, complemented by umbilical cord blood and placental tissue for the perinatal period. Information on prenatal stress is usually not available in human postmortem brain studies which rely on postmortem tissue from brain banks. In animal studies, a wider range of tissues, including brain, are accessible. This provided further insight into the effects of prenatal stress on epigenetic regulation [2, 14, 24, 31, 33, 62], but translating these findings to humans entails challenges.

### Candidate gene studies

Studies in humans often focused on candidate genes involved in the HPA-axis (see Table 1). The HPA-axis is a main regulator of stress response, and it has been shown that maternal stress during pregnancy can lead to long-term effects on HPA-axis function and stress-related outcomes in the fetus [25, 54]. One of the most studied HPA-axis candidate genes is *NR3C1* encoding the glucocorticoid receptor (*GR*), which itself is important for the negative feedback loop of the HPA-axis [22]. Interest in DNAm of *NR3C1* was initiated by studies in rats: *nr3c1* exon 1F methylation in the brain of offspring varied by maternal care and had relevance for *GR*-expression [104]. In human blood samples from children, DNAm of the *NR3C1* promoter was associated with maternal prenatal stress experiences, such as intimate partner violence [79] and exposition to the Tutsi genocide [76]. In a meta-analysis of seven studies across different tissues, a significant correlation between prenatal maternal psychosocial stress and offspring DNAm at a CpG site located in the exon 1F of *NR3C1* was observed [71, 72]. Furthermore, Turecki and Meaney [99] evaluated both animal and human studies and found an association of prenatal stress and increased methylation of *NR3C1*. Another systematic review assessed associations among maternal prenatal stress (defined as traumatic life events, stressful situations and perception of stressors, or the resulting phenotypes of stressors) and DNAm among commonly studied HPA-axis candidate genes (*11BSHD2, OXTR, SLC6A4, CRH, CRHBP, FKBP5*) in infants less than one year old [93]. The genes examined in this study are commonly considered candidates implicated in HPA-axis regulation. *FKBP5* is involved in the termination of the stress response by regulating GR sensitivity [107]. *11BHSD2* is known as an important placental gene encoding an enzyme that catalyzes cortisol into cortisone and thus protects the fetus from excess cortisol exposure [85]. *CRH* and *CRHBP* are related to the production and release of cortisol by the pituitary gland as part of HPA-axis functioning [44]. The serotonin receptor gene *SLC6A4* is implicated in the serotonergic modulation of the HPA-axis [77], and the oxytocin receptor gene, OXTR, is assumed to play a role for HPA-axis inhibition [35, 65]. The authors in [93] reported evidence for a link between prenatal stress and *NR3C1* methylation, with more severe stressors showing stronger associations with infant DNAm as compared to stress-response phenotypes in the mothers. In another study by Kertes et al., widespread effects on DNAm in neonatal cord blood and placenta were reported among the *CRH, CRHBP, NR3C1* and *FKBP5* gene following prenatal traumatic war-related stress exposure, but the associations differed between tissues and were weaker for chronic stress compared to trauma [43].

**Table 1:** Overview of referenced candidate genes studies investigating the association between DNAm and prenatal stress

References	Candidate genes	Method DNAm	Sample Size	Exposure, exposure sample size and timing (if available)	Outcome and timing	Tissue	Age and sex (if available)	Result*
[43]	*CRH, CRHBP, FKBP1, NR3C1*	450 K array	24	Prenatal psychosocial stress; 31% with 0–1 chronic stressors; 69% with 2–18 chronic stressors	DNAm at birth	Cordblood,placenta	Newborns; 54% male	Effects on cord blood DNAm in *CRH* and *NR3C1*, effects on placenta DNAm in all four tested genes
[60]	*FKBP5, HSD11B2, NR3C1*	450 k array	59	Perceived maternal prenatal stress scale	DNAm at birth	Placenta	Newborns; 49% male	Higher perceived stress associated with higher DNAm
[63]^a^	*NR3C1,* *SLC6A4*	Next-generation Sequencing (NGS)	283	Pandemic lockdown; *n* = 81 in 1st trimester, *n* = 84 in 2nd trimester, *n* = 118 in 3rd trimester	DNAm 6–24 h after birth	Buccal	Newborns; 50% male	Higher methylation if exposed to pandemic lockdown during 2nd or 3rd trimester
[71, 72]	*NR3C1*	Different	977 (meta-analysis)	Maternal chronic psychosocial stress during pregnancy	DNAm in children	Different	Newborns; one study included adolescents at mean age of 14 years	Significant correlation of DNAm and psychosocial stress
[76]	*NR3C1,* *NR3C2*	Pyro-sequencing	50	Exposure to Tusti genocide during pregnancy; *n* = 25 offspring of mothers exposed to Tutsi genocide during pregnancy, *n* = 25 unexposed	DNAm in children	Blood	Adolescents aged 17–18 years, 48% male in exposed, 36% male in unexposed	Higher DNAm in *NR3C1* in children whose mothers had been exposed
[78]^a^	*SLC6A4*	NGS	108	Covid19-related prenatal stress score in 3rd trimester	DNAm 6–24 h after birth	Buccal	Newborns; 49% male	Hypermethylation in 3rd trimester
[79]	*NR3C1*	NGS	24	Intimate partner violence (IPV) in pregnancy; *n* = 8 with IPV, *n* = 16 without IPV	DNAm in children	Blood	Children 10–19 years; 33% male	Positive relationship between DNAm and IPV in pregnancy
[93]	*11HSDB2, CRH,* *CRHBP,* *FKBP5,* *NR3C1,* *OXTR, SLC6A4*	Different	Systematic review	Maternal stress in pregnancy	DNAm in infants	Different	Children aged below 12 months	Strongest association between most severe stressors and infant DNAm
[99]	*NR3C1*	Different	Systematic review	Early life adversity, maternal psychosocial stress, parental stress	DNAm in offspring	Different	Children from birth to 18 years of age	Increased DNAm in exposed children
[106]	*FKBP5*	Pyro-sequencing	31	Holocaust experience, not only during pregnancy; *n* = 22 whose parents experienced holocaust, *n* = 9 whose parents did not experience holocaust	DNAm in offspring	Blood	Offspring; mean age 46–47 years; 27% male in exposed, 11% male in unexposed	Lower *FKBP5* DNAm in offspring of holocaust survivors

Studies are listed in alphabetical order; studies are cross-sectional if not stated otherwise

^*^significance was defined differently throughout the studies

^a^note that both studies used overlapping datasets

DNAm changes in *FKBP5* were also reported in blood samples of adult offspring of holocaust survivors [106]. Furthermore, perceived maternal stress was associated with increased placental DNAm of both *HSD11B2* and *FKBP5* and lower fetal heart rate-movement coupling, which is an indicator for fetal central nervous system development [60].

*NR3C1* as well as *SLC6A4* methylation has also been linked to maternal pandemic stress [63], interestingly a heightened sensitivity to epigenetic regulation could only be observed in mothers and their offspring exposed to lockdown in the second or third pregnancy trimester, but not in dyads exposed in the first trimester. In the same cohort and in accordance with this finding, Provenzi et al. [78] reported higher *SLC6A4* methylation in children whose mothers reported pandemic stress in the last trimester.

### Epigenome-wide association studies

As an alternative to candidate gene approaches, epigenome-wide association studies (EWAS) of DNAm, assessing the association between all CpG sites available on DNAm arrays and a trait of interest, have gained popularity during the last decade (see Fig. 1A and Table 2). Large sample sizes are necessary to detect the usually small effect sizes of single CpGs; hence, EWAS are usually conducted in large consortia, such as the **P**regnancy **A**nd **C**hildhood **E**pigenetics (PACE) consortium [27], where association results of single cohorts are pooled. While several epigenome-wide significant CpGs for maternal risk factors such as smoking (top hit in *AHRR* [42]) and maternal body mass index (BMI) at the beginning of pregnancy (top hit in *VPR2* [91]) could be detected, no significant associations for maternal alcohol intake [90] and maternal anxiety [82] have been reported.

**Table 2:** Overview of referenced epigenome-wide studies investigating the relationship between DNAm and prenatal stress

References	Candidate genes	Method DNAm	Sample Size	Exposure, exposure sample size and timing (if available)	Outcome and timing	Tissue	Age and sex (if available)	Result*
[11]	EWAS	450K array	207	Maternal lifetime exposure to stress score assessed in 2nd trimester	DNAm at birth	Placenta	Newborns; 52% male	112 associated CpG sites (FDR 0.05)
[37]	EWAS	EPIC v1 array	8	Maternal Covid-19 infection; *n* = 4 exposed, *n* = 4 unexposed	DNAm in infants	Buccal	Infants aged 3 months; 88% male	2,678 associated CpG sites (nominal p-value < 0.05)
[42]	EWAS	450K array	6685	Maternal smoking in pregnancy; *n* = 1,646 exposed to any smoking in pregnancy, *n* = 5,039 unexposed	DNAm at birth	Cordblood	Newborns	6,073 associated CpG sites (FDR 0.05); 254 CpGs significantly associated with gene expression (FDR 0.05)
[47]	EWAS	EPIC v1 array	44	Exposure to Covid-19 pandemic in utero; *n* = 32 exposed, *n* = 12 unexposed	DNAm in infants	Buccal	Infants mean age 5 weeks; 56% male exposed; 58% male unexposed	675 associated CpG sites (FDR 0.05)
[80]	EWAS	450K array	1740	Prenatal maternal exposure score	DNAm at birth	Cordblood	Newborns; 51% males	No significantly associated CpG sites after multiple testing correction
[82]	EWAS	450K array or EPIC v1 array	7243	Maternal anxiety score during pregnancy	DNAm at birth	Cordblood	Newborns	No significantly associated CpG sites after multiple testing correction
[89]	EWAS	EPIC v1 array	114	Prenatal maternal perceived stress	DNAm at birth	Saliva	Newborns; 54% male	One associated CpG site (FDR 0.05)
[91]	EWAS	450K array	9340	Pre-Pregnancy maternal BMI	DNAm at birth	Cordblood	Newborns	9,044 associated CpG sites (Bonferroni correction)
[90]	EWAS	450K array	3075	Alcohol consumption in pregnancy; *n* = 1147 exposed, *n* = 1928 unexposed	DNAm at birth	Cordblood	Newborns	No significantly associated CpG sites after multiple testing correction
[98]	EWAS	450K array	930	Maternal famine exposure during pregnancy; *n* = 73 exposed in weeks 1–10 of gestation, *n* = 123 exposed in weeks 11–20 of gestation, *n* = 143 exposed in weeks 21–30 of gestation, *n* = 128 exposed in weeks 31 up to delivery, n = 463 unexposed	DNAm in adults	Blood	Adults; mean age 59; 46% male	4 associated CpG sites with exposure in gestational weeks 1–10 after multiple testing correction, no associated CpG sites with exposure in later gestation
[100]	EWAS	EPIC v1 array	16	Maternal Covid-19 infection; *n* = 8 exposed, *n* = 8 unexposed	DNAm at birth	Cordblood	Newborns	119 associated CpG sites (FDR 0.20)

Studies are listed in alphabetical order; studies are cross-sectional if not stated otherwise

^*^significance was defined differently throughout the studies

In general, EWAS are often underpowered, effect sizes are usually small [93, 96] and, up to now, EWAS results for prenatal stress have been rather inconsistent. Rijlarrsdam et al. performed both an individual EWAS and a meta-analysis in cord blood, which revealed no epigenome-wide association between prenatal maternal stress and DNAm [80]. However, maternal life stress was associated with placental DNAm patterns of genes associated with endocytosis (i.e., *SMAP1, ANKFY1*), tight junctions (i.e., *EPB41L4B)*, and metabolic pathways (i.e., *INPP5E, EEF1B2*), implicating roles for early embryo development [11]. Finally, a recent EWAS revealed associations between newborn epigenome-wide DNAm levels measured in saliva and chronic psychosocial stress experienced by the mother during pregnancy [89]. The associated genes including *CSMD1, DAXX* and *ARL4D* are relevant for neuronal, immune and endocrine homeostasis.

An EWAS on pandemic stress has been conducted confirming the role of *NR3C1* as marker of prenatal stress [47]. Prenatal Covid-19 infection itself has been associated with DNAm changes in the offspring, albeit with very small sample sizes (8–16 samples): Hill et al. (R. A. [37] identified CpG sites in *AFAP1* as well as in *GAREM2* while [100] found associated CpGs in stress-response pathways.

EWAS also revealed that the timing of the prenatal stressor seems to be important. Using the quasi-experimental setting of the Dutch Famine, CpGs measured in adult whole blood were related to prenatal famine exposure during the first 10 weeks of gestation, but were not associated to stress exposure later in gestation. Interestingly, the identified CpGs were linked to genes with roles in growth, differentiation and metabolism (*FAM150B, SLC38A2, PPAP2C* and *OSBPL5/MRGPRG*) [98]*.*

### Epigenetic clocks

Since the development of the first epigenetic clocks in 2013 [32, 39], measurements of epigenetic aging constitute another method to investigate relationships between environmental influences and epigenetics (see Table 3). Epigenetic clocks aim to estimate the biological age of an individual from DNAm at specific CpGs that have previously been related to aging (see [40, 81] for an overview). Higher DNAm age as compared to chronological age is referred to as ‘age acceleration,’ lower DNAm age as compared to chronological age is referred to as ‘age deceleration,’ and this ticking rate of an epigenetic clock has been associated with age-related diseases [105]. Although there are no human studies to date which focus specifically on prenatal stress and epigenetic aging in fetal tissues, prenatal adverse environment was linked to epigenetic age deceleration in cordblood [73]. Moreover, antenatal depressive symptoms were related to age deceleration in cordblood [94], but prenatal selective serotonin reuptake inhibitors (SSRIs) use could significantly contribute to this association [57]. In addition, prenatal maternal anxiety predicted child epigenetic age acceleration, however only in males [55]. This underscores that sex can be an important modifier in DNAm studies. Furthermore, we observed that gestational epigenetic aging is not related to more favorable or unfavorable factors in a clear direction in placenta and cord blood, and epigenetic aging patterns are tissue specific [23].

**Table 3:** Overview of referenced studies investigating the association between epigenetic age and prenatal stress

References	Candidate genes	Method DNAm	Sample Size	Exposure, exposure sample size and timing (if available)	Outcome and timing	Tissue	Age and sex (if available)	Result*
[55]	Epigenetic age, longitudinal study	EPIC v1 array	505	Prenatal maternal anxiety, externalizing problems in offspring	DNAm in infants and children	Buccal	Children; aged 3, 9, 48 months, 6 and 10 years	Prenatal maternal anxiety associated with age acceleration; age acceleration associated with externalizing problems in boys
[73]	Epigenetic age	EPIC v1 array	60	Cerebroplacental ratio (CPR) in 3rd trimester	DNAm at birth	Cordblood	Newborns	Decreased epigenetic age acceleration in children associated with decreased CPR
[94]	Epigenetic age	450K array	407	Antenatal depressive symptoms	DNAm at birth	Cordblood	Newborns; 53% males	Decreased epigenetic age acceleration in children associated with maternal depressive symptoms

Studies are listed in alphabetical order; studies are cross-sectional if not stated otherwise

^*^significance was defined differently throughout the studies

## Are prenatal stress-associated epigenetic changes related to child health outcomes?

In the previous section, we discussed associations between prenatal stress and epigenetic changes, focusing on DNAm. These epigenetic alterations were often related to HPA-axis function and have the potential to prime the child, for example regarding long-term stress responsiveness. Hence, prenatal stress may increase the risk for altered physiological and psychiatric outcomes in the offspring through epigenetic mechanisms (see Fig. 1B and Table 4).

**Table 4:** Overview of referenced studies investigating the association between DNAm and child outcome

References	Candidate genes	Method DNAm	Sample Size	Exposure, exposure sample size and timing (if available)	Outcome and timing	Tissue	Age and sex (if available)	Result*
[1]	*HSD11B2,* *NR3C1*	Pyro-sequencing	372	Infant neurobehavior (NICU Network Neurobehavioral Scales (NNNS))	DNAm at birth	Placenta	Newborns; 50% male	Low *NR3C1* DNAm and high H*SD11B2* DNAm associated with lower excitability scores; high *NR3C1* DNAm and low *HSD11B2* DNAm associated with asymmetrical reflexes; high DNAm associated with higher habituation scores
[17]	*HSD11B2,* *NR3C1*	Pyro-sequencing	482	Prenatal maternal anxiety and depression	DNAm and internalizing problems	Placenta	Newborns; 48% male	Positive correlation between *NR3C1* DNAm and internalizing problems
[68]	*NR3C1*	Pyro-sequencing	82	Depressed/ anxious mood in 3rd trimester	DNAm in newborns and cortisol response at 3 months	Cordblood	Newborns; 44% male	Hypermethylation in *NR3C1* which was also associated with increased HPA stress responsiveness at 3 months
[13]^a^	Candidate genes from established Type-1 and -2 diabetes mellitus pathways	450K array	31	Maternal stress during exposure to Quebec ice storm	DNAm, child BMI	Blood	Children; mean age 13 years	Significant negative mediation of DNAm of the effect of objective prenatal maternal stress on central adiposity and BMI
[12]^a^	Candidate genes from established Type-1 and -2 diabetes mellitus pathways	450K array	30	Maternal stress during exposure to Quebec ice storm	DNAm, C-peptide secretion in response to an oral glucose tolerance test	Blood	Children; mean age 13 years	Significant mediation of DNAm of the relationship between maternal cognitive appraisal of a natural disaster in pregnancy and C-peptide production in adolescent offspring
[46]	*FKBP5*	Pyro-sequencing	76	Childhood trauma; *n* = 30 exposed, *n* = 46 unexposed; glucocortiocoid receptor sensitivity	DNAm in adults	Blood	Adults; mean age 41 years	Allele-specific, childhood trauma–dependent DNA demethylation in functional glucocorticoid response elements of *FKBP5* linked to dysregulation of the stress hormone system and a global effect on the function of immune cells and brain areas associated with stress regulation
[52]	*HSD11B2*	Pyro-sequencing	185	NNNS	DNAm	Placenta	Newborns; 45% male	Increasing DNAm associated with reduced scores of quality of movement; increased DNAm associated with lower expression of *HSD11B2*
[74]	*FKBP5*	Pyro-sequencing	509	NNNS	DNAm	Placenta	Newborns; 49% male	Infants in the highest quartile of *FKBP5* DNAm with increased risk of NNNS high arousal compared to infants in the lowest quartile
[75]	*ADCYAP1R1,* *FKBP5,* *HSD11B2,* *NR3C1*	Pyro-sequencing	537	NNNS	DNAm	Placenta	Newborns; 49% male	DNAm patterns of glucocorticoid response genes associated with adaption to postnatal stress among healthy populations of infants exposed to low-to-moderate prenatal stress

Studies are listed in alphabetical order; studies are cross-sectional if not stated otherwise

^*^significance was defined differently throughout the studies

^a^note that both studies used overlapping datasets

Again, the glucocorticoid receptor gene is the candidate gene with the strongest evidence. For example, increased DNAm at a CpG in *NR3C1* in cord blood after prenatal exposure to maternal depressed mood was related to HPA-axis stress reactivity of the child at three months of age [68]. Using placental samples, Conradt et al. [17] identified a relationship between *NR3C1* DNAm with infant quality of movement and attention. In a systematic review on DNAm of the glucocorticoid receptor gene the link between early life adversity, hypermethylation and impaired HPA-axis functioning, which may predispose individuals to psychiatric conditions, was supported [71, 72]. Additionally, it was suggested that placental DNAm of *NR3C1* and *HSD11B2* may jointly influence distinct domains of newborn neurobehavior [1].

Effects of maternal reports of depression or anxiety during pregnancy were related to neurobehavioral outcomes in the newborns and placental DNAm in *NR3C1* and *11BHSD2* [17, 52] as well as in *FKBP5* [74]. Moreover, Klengel et al. [46] provide evidence for *FKBP5* DNAm mediating the combined effect of early trauma exposure and a genetic polymorphism in *FKBP5* on the risk of developing stress-related psychiatric disorders using peripheral blood in adults. In another study, placental DNAm patterns of four candidate glucocorticoid response genes (*NR3C1, HSD11B2, FKBP5 and ADCYAP1R1*) were related to risk of neurobehavioral adversity, i.e., different ability to adapt to stress in the postnatal environment, in a healthy population of infants [75].

Studies on natural disasters also support the mediating role of DNAm on the association between prenatal maternal stress exposures and the child’s metabolic outcomes and immune system: Cao-Lei et al. [13] report that DNAm of selected genes in the type 2 diabetes pathway mediated the association of objective prenatal maternal stress due to the Quebec ice storm and adiposity in teenage children. Adding to this, the relationship between objective hardship experienced by the mothers and C-peptide secretion in adolescent children was also mediated by blood DNAm [12].

This relationship between stress and trauma exposure of parents and a greater risk of psychopathology among children, accompanied by epigenetic modification, is supported by the concept of intergenerational transmission brought forward by Bowers et al. [10], although the etiology of effects in humans is impeded by methodological constraints. Congruently, Monk et al. [61] state that there is evidence for an association between maternal prenatal distress and both fetal and infant developmental trajectories where epigenetic mechanisms may serve as a biological link mediating these effects, but influences of the postnatal environment must be carefully considered to further elucidate these pathways.

The postnatal environment is not only important to consider on a methodological level as a confounder, but should also be taken into account to understand the demonstrated relationships between prenatal stress and child outcomes from an evolutionary perspective and including the match/mismatch hypothesis. Epigenetic mechanisms can fine-tune gene expression to allow the organism to adapt to the environment [6]. Accordingly, there should not be a one-sided perspective of stress causing vulnerability for diseases. There is also an adaptive side of epigenetic modifications following prenatal exposures, preparing the developing organism for later environment. For example, a study by Zhang et al. [108] supports the idea that a moderate amount of normative prenatal stress may buffer the impact of traumatic prenatal stress, in this case caused by Superstorm Sandy, on placental gene expression. And Serpeloni et al. [87] showed that children had better mental health, when pre- and postnatal environment matched, i.e., there were less psychiatric problems among children who experienced intimate partner violence prenatally and postnatally compared to children who experienced this only postnatally. These studies demonstrate the possibility of adaptive programming following early experiences. In summary, changes after prenatal exposure to stress could favor developmental adjustment accomplished by epigenetic alterations which should not only be seen as detrimental, but also evolutionarily adaptive to some extent. Finally, individual differences in the sensitivity to early programming should be considered [64].

## Summary

In conclusion, there is some evidence from candidate gene studies as well as from EWAS that prenatal stress is associated with changes in DNAm and that these changes could also have impact on child health outcomes. When evaluating these findings, it is essential to be aware of the limitations and generalizability of described associations. First of all, DNAm can vary by ancestry [41] and also the genome plays an important role—allele-specific DNAm patterns [58, 92] as well as meQTLs (methylation quantitative trait loci, [59]) have been reported. By design, EWAS focus on main effects of specific traits, such as prenatal stress, on DNAm but do not include gene-environment interactions (GxE). However, the child’s genetic susceptibility might be of importance as it has been shown that GxE effects explain the majority of variation of DNAm in the pre- and postnatal context [19, 20, 97]. Moreover, child sex and age have been related to both DNAm [9] and the stress exposure itself [56, 95] and stratified analyses might be beneficial to uncover DNAm signatures in subgroups.

Most epigenetic studies focused on main effects of prenatal stress on DNAm. However, it should be noted, that other important factors need to be taken into account to disentangle the path from prenatal stress to DNAm and outcome in the offspring. On the one hand, prenatal stress and its consequences on maternal physiology themselves can be mediated by factors such as diet, sleep or exercise [21]. On the other hand, effects of prenatal stress on the offspring can be enhanced by postnatal stress but also be reversed by postnatal supportive environment [66]. Only few studies have looked into this relationship on the level of DNAm.

Moreover, not only the type of stressor but also the timing of the stress exposure during pregnancy can play an important role, considering different susceptible periods during fetal development (Van den [102].

Tissue specificity is another major challenge, for example relevant whenever translating animal research to humans and explaining incongruencies between results of different studies. Epigenetic mechanisms leading to differential gene expression are a central part of cell development and differentiation, and thus they vary to some extent between developmental stages and tissues [8]. Hence, findings based on DNAm patterns in one tissue cannot be directly transferred to DNAm effects in other tissues.

Finally, only looking into DNAm depicts only a limited picture as DNAm itself can have further consequences on gene expression. Of the investigated studies, only one reported a significant association of identified CpG sites with gene expression [52] and often studies did not evaluate gene expression in their samples at all.

### Implications for future studies

It has been shown, both in animal and human studies, that prenatal stress can be related to epigenetic changes, and the period from conception to early childhood seems to be the most critical one [51]. However, robust, replicated findings are still scarce. What could be the “mission” for future studies? Four main points are important here: 1) the timing and intensity of the stressor, 2) the definition of the stressor itself, 3) in which tissue an effect was observed and 4) a longitudinal study design. The prenatal period is a dynamic and developmentally important phase making it very interesting but also very complex to study. Most studies used a cross-sectional design where the presence of a prenatal stressor was associated to DNAm levels at one specific timepoint, usually in perinatal tissues at birth. To be able to reflect these dynamic changes in the prenatal period, however, ideally, longitudinal measurement should be assessed. This refers to the timing and course of prenatal stress itself. Furthermore, if prenatal stress is associated with epigenetic changes, a very important question is if this also leads to long-lasting changes in DNAm signatures and in child development or might be moderated by postnatal environment. Hence, not only follow-up during pregnancy but also after birth is essential, including not only repeated phenotypes on child outcome but also repeated DNAm measures, and ideally also gene expression, throughout childhood and beyond.

Furthermore, it still remains inconclusive if the existence of *any* prenatal stressor or rather of specific prenatal stressors are implicated in DNAm changes. The operationalization of prenatal stress must therefore be taken into account. The definition of stress varies between studies and different stressors could have distinct effects. Hence, refined measurement and definition of prenatal stress and its timing, ideally also incorporating genomic information, will provide further opportunities to enhance our understanding of the biological embedding of prenatal stress exposures.

All of these points potentially contribute to heterogeneous results reported in the literature and are momentous when interpreting individual findings. Indeed, the role of the reported associations as potential causal mechanisms is mostly not clear, especially in humans, where we rely on peripheral tissues and often retrospective designs. Importantly however, they could still serve as biomarkers for those processes that in the end lead to disease trajectories.

To date, most studies have been focusing on DNAm in cord blood, while only some used placental tissue. The placenta is heterogeneous and challenging to study, however, given its importance for fetal development [28] and its possible role in early neurodevelopment [101], it should not be neglected and future studies should also look more closely into placental DNAm.

Given that the mentioned limitations are accounted for as far as possible, DNAm biomarkers have the potential to improve our understanding of prenatal programming. Enlightening these processes and their long-term consequences will also enhance our knowledge on children’s health care [88].

## Data Availability

Not applicable.

## Abbreviations

BMI: Body mass index
DNAm: DNA methylation
DOHaD: Developmental origins of health and disease
EWAS: Epigenome-wide association studies
GC: Glucocorticoid
GR: Glucocorticoid receptor
HPA-axis: Hypothalamic–pituitary–adrenal-axis
PACE consortium: Pregnancy and childhood epigenetics consortium
PAR: Predictive adaptive response
SSRI: Selective serotonin reuptake inhibitor
meQTL: Methylation quantitative trait locus
